# The Role of Vitamin C in Selected Autoimmune and Immune-Mediated Diseases: Exploring Potential Therapeutic Benefits

**DOI:** 10.3390/ijms26199375

**Published:** 2025-09-25

**Authors:** Martyna Mochol, Lukasz Jablonowski, Andrzej Pawlik, Joanna Rasławska-Socha, Agnieszka Chamarczuk, Mariusz Lipski, Małgorzata Mazurek-Mochol

**Affiliations:** 1 Department of Periodontology, Pomeranian Medical University in Szczecin, Powstańców Wlkp 72, 70-111 Szczecin, Poland; 2 Department of Restorative Dentistry, Periodontology and Endodontology, University Medicine Greifswald, Walther-Rathenau-Str. 42a, D-17475 Greifswald, Germany; 3 Department of Physiology, Pomeranian Medical University in Szczecin, Powstańców Wlkp 72, 70-111 Szczecin, Poland; 4 Department of Preclinical Conservative Dentistry and Preclinical Endodontics, Pomeranian Medical University in Szczecin, Powstańców Wlkp 72, 70-111 Szczecin, Poland

**Keywords:** vitamin C, autoimmune disease, immune-mediated disease, nutrition

## Abstract

Autoimmune diseases are characterized by immune response dysregulation against self-components, leading to chronic inflammation and tissue damage. Vitamin C (VitC), a water-soluble vitamin with established functions in antioxidant defence and collagen synthesis, has also been of interest based on its potential immunomodulatory effects. This review discusses the role of VitC in the course and progression of (A) autoimmune diseases (multiple sclerosis, rheumatoid arthritis, Sjögren’s disease, type 1 diabetes, Hashimoto’s thyroiditis, pernicious anaemia, antiphospholipid syndrome), (B) other immune-mediated diseases (Crohn’s disease, periodontitis), and (C) Alzheimer’s disease, a neurodegenerative disorder with autoimmune features. Results from clinical, observational, and experimental trials show that VitC deficiency is common in many of these diseases and may contribute to increased oxidative stress and immune disequilibrium. Supplementation has been associated with improved antioxidant levels, control of inflammatory mediators, and, in some cases, clinical outcomes like disease activity decrease or symptom load. Although findings vary across conditions and few large, randomized trials are available, the overall evidence indicates that maintaining good VitC status can be useful in maintaining immune homeostasis and reducing inflammation. VitC should be viewed as an adjunct to be employed safely, perhaps and ideally within larger treatment regimens, but not in place of effective therapies. Further research, including large-scale clinical trials, will be required to determine more clearly optimal dosing, timing of treatment, and patient population most likely to benefit. By integration of current knowledge, this review recognizes both promise in VitC for treatment of autoimmune/immune-mediated disease and promise in its potential use within future treatment regimens.

## 1. Introduction

The pathogenesis of autoimmune diseases involves dysregulation of the immune system, resulting in the destruction of healthy tissues and the production of autoantibodies. While the exact causes remain elusive, recent evidence indicates that nutritional factors, such as VitC, play a role in the course and management of immune system diseases [1].

**Figure 1:**
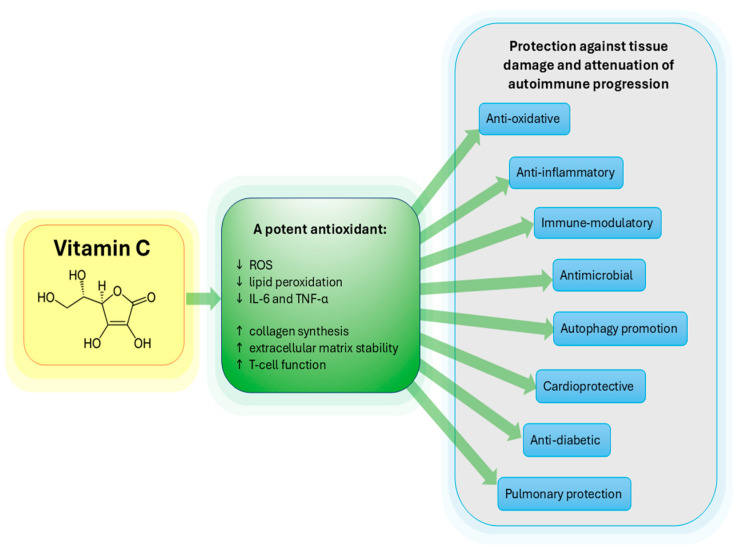
Role of Vitamin C in various physiological processes.

VitC, also known as ascorbic acid, is a water-soluble vitamin with well-established roles in various physiological processes (Figure 1). Beyond its recognized role as an antioxidant and cofactor in collagen synthesis, VitC has garnered attention for its immuno-modulatory properties and potential therapeutic benefits in the immune system diseases [1,2]. Through its influence on immune cells, cytokine regulation, and antioxidant activity, VitC may have profound implications for modulating the immune response and mitigating the progression and severity of autoimmune diseases.

Immunomodulation is a key mechanism by which VitC may exert its effects in autoimmune diseases. VitC can modulate immune cell function, enhancing immune responses while limiting excessive activation [1]. By promoting the development and maturation of immune cells, such as T cells, B cells, and natural killer cells, VitC may support the immune system’s ability to mount an appropriate response against pathogens or abnormal cells, while also preventing immune dysregulation [1,3].

In addition to its direct effects on immune cells, VitC plays a crucial role in regulating the production and activity of cytokines, which are central mediators of inflammation and immune responses. VitC can modulate the balance between pro- and anti-inflammatory cytokines, helping achieve a balanced immune response. By suppressing the production of pro-inflammatory cytokines, such as interleukin-6 (IL-6) and tumour necrosis factor-alpha (TNF-α), while promoting the synthesis of anti-inflammatory cytokines, including IL-10, VitC may help mitigate excessive inflammation and tissue damage in autoimmune diseases [1,4].

Oxidative stress and chronic inflammation are interconnected processes implicated in the pathogenesis of autoimmune diseases. VitC’s potent antioxidant properties enable it to scavenge reactive oxygen species (ROS) and neutralize free radicals, thereby reducing oxidative stress and limiting inflammation [1,5]. By maintaining a redox balance, VitC may help protect cells from oxidative damage and modulate inflammatory signalling pathways, potentially attenuating the inflammatory cascade seen in autoimmune diseases. High-dose intravenous VitC has been reported to reduce pain, fatigue, and cognitive impairment in observational studies in conditions linked to oxidative stress [6,7,8,9,10].

While the immunomodulatory and antioxidant effects of VitC are promising, it is essential to consider individual variations, disease-specific factors, and potential interactions with existing treatments. The optimal dosage, duration, and timing of VitC supplementation in autoimmune diseases warrant further investigation to ensure maximum therapeutic benefits [1].

VitC has been investigated as a potential adjunct in the management of autoimmune diseases. However, further research is needed to elucidate the optimal strategies for incorporating VitC into comprehensive treatment approaches. By unravelling its full potential, future research could clarify for improved outcomes and enhanced quality of life for individuals living with autoimmune conditions.

The role of VitC in autoimmune diseases has been a topic of interest among researchers for many years. The findings indicate that use of VitC as part of the conventional treatments for autoimmune disease often comes with significant side effects and limited efficacy. Therefore, exploring the potential of VitC supplementation as an alternative or adjunctive therapy is of the utmost importance. While VitC is generally considered safe and well tolerated, high doses may cause gastrointestinal upset, kidney stone formation, and interactions with certain drugs, potentially compromising their efficacy or safety. Therefore, a comprehensive understanding of the potential risks and benefits of VitC supplementation is essential before recommending its use in any autoimmune disease management.

**Figure 2:**
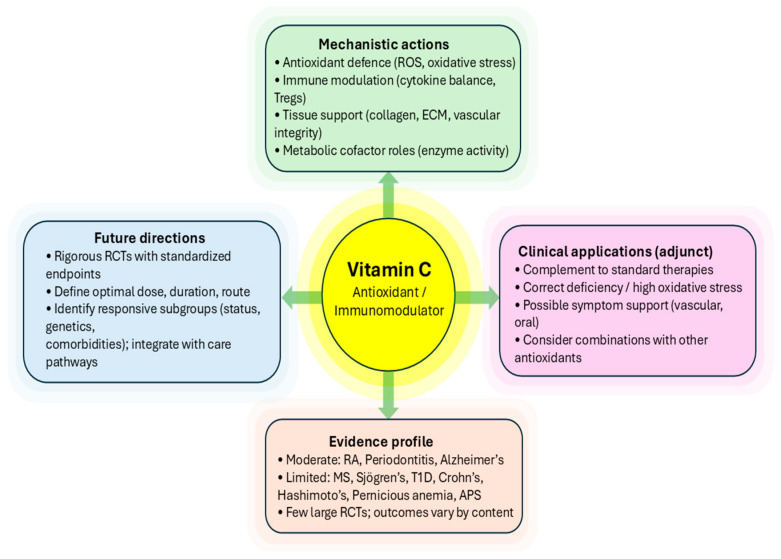
Potential roles and applications of Vitamin C in autoimmune and immune-mediated disease management.

In light of these considerations and because of the vastness of this subject, this review aims to review the role of VitC in the course and progression of selected autoimmune and immune-mediated diseases. It explores the evidence regarding the effectiveness of VitC supplements in treating the disease and the potential side effects associated with their use. By addressing these questions, this review seeks to contribute to the existing knowledge on the role of VitC in autoimmune diseases and summarise therapeutic strategies for their management (Figure 2).

Classification used in this review—(A) confirmed autoimmune diseases (multiple sclerosis (MS), rheumatoid arthritis (RA), Sjögren’s disease (SD), type 1 diabetes (T1D), Hashimoto’s thyroiditis (HT), pernicious anemia (PA), antiphospholipid syndrome (APS)), (B) immune-mediated inflammatory diseases (Crohn’s disease (CD), periodontitis (PD)), and (C) a neurodegenerative disorder with autoimmune features (Alzheimer’s disease (AD)).

## 2. Multiple Sclerosis

There is limited number of studies on the association between MS and VitC. However, the available data have revealed that patients with MS have decreased VitC serum levels compared with healthy individuals [11,12]. Additionally, the elevated oxidative burden and increased lipid peroxidation in patients with MS during relapse has been highlighted in more recent reviews of antioxidant therapies, where VitC is noted to contribute to reductions in oxidative stress markers [13].

The mechanisms underlying the therapeutic benefits of VitC are based on its antioxidant properties [14]. VitC plays a role in maintaining the integrity and function of processes in the central nervous system, while its deficiency can lead to cerebral hemorrhage and death in mice [15]. VitC has shown possible therapeutic effects in the treatment of MS [14]. Clinical evidence suggests that VitC supplementation in MS patients may reduce relapse frequency and improve MRI lesion profiles [16], and reviews emphasise that VitC is part of multi-antioxidant strategies that help attenuate oxidative stress [13].

A number of studies have suggested that VitC may have a protective effect against MS, and that its antioxidant properties may help reduce oxidative stress [17,18,19]. These findings suggest that VitC is somehow involved in the pathophysiology of MS. In another study [20], the authors reported that cerebrospinal fluid (CSF) from patients with secondary progressive multiple sclerosis (SPMS) contained more VitC than CSF from patients with primary progressive multiple sclerosis (PPMS). This finding suggests that the brain’s capacity to retain VitC may play a protective role to delay the worsening of symptoms for both courses of the disease.

The antioxidant properties of VitC may be used in the clinical treatment of MS. In addition to its antioxidant properties, VitC enhances the formation of myelin [21], and it may serve as an adjuvant in stem cell therapies [20]. Although VitC may have a protective effect against MS, more research is needed to better understand its role as a treatment for this autoimmune disease. Additionally, due to the limited evidence available, it is not known whether VitC is safe to use in high doses [22]. Therefore, if considering the use of VitC for MS, it is important to consult a doctor first to consider the potential risks and benefits of this supplement.

## 3. Rheumatoid Arthritis

Both clinical and experimental studies suggest that VitC may influence the development and progression of the disease. Data from large population cohorts show that people with higher serum VitC concentrations are less likely to have RA. For example, an analysis of more than 12K adults from the NHANES survey found an inverse relationship between VitC levels and RA prevalence [23]. Similar results have been reported in dietary studies, where a higher intake of antioxidants, including VitC, was linked to a lower risk of RA [24]. Another study confirmed that individuals with lower VitC consumption tended to follow more pro-inflammatory diets and had more active disease [25].

Experimental models help explain these associations. In collagen-induced arthritis, supplementation with VitC improved disease outcomes by restoring a healthier gut microbiota profile and reducing systemic inflammation [26]. Other work has shown that ascorbic acid can limit the generation of autoreactive plasma cells, which in turn reduces autoantibody production and joint damage. This effect appears to be mediated through the inhibition of STAT3 signalling [27].

Clinical data, although still limited, point in the same direction. A small, randomized trial combining VitC and VitE with standard therapy showed improvements in oxidative stress markers and some clinical symptoms [28]. A systematic review and meta-analysis of supplementation studies also found that VitC lowers circulating levels of IL-6, one of the key pro-inflammatory cytokines in RA [29]. Broader nutritional interventions add to this evidence: for example, in a trial of multigrain supplementation, patients achieved better disease control and reduced inflammation, and increased VitC intake was one of the factors contributing to the results [30].

Several reviews now emphasize the relevance of VitC in modulating oxidative stress and immune responses in RA [26,31]. Clinical trials specifically targeting VitC supplementation are still in progress, such as the study registered under NCT04036110, which may provide clearer evidence on dosage and long-term benefits [32].

Another study shows that the WNT signalling pathway can play a key part in RA. Patients with these conditions have higher levels of DKK1, Wnt5a, and β-catenin in their blood. In RA, DKK1 levels track with disease activity and bone breakdown, while β-catenin links with inflammation markers like IL-6 [33]. VitC has not been directly studied with these WNT markers in RA, but there are some interesting clues from other systems. In bone, VitC switches on the WNT/β-catenin/ATF4 pathway, which helps build bone by boosting osteoblasts and slowing down osteoclasts [34]. In immune cells, VitC also calms inflammation by turning on β-catenin signalling through changes in GSK3β [35]. On top of that, VitC’s strong antioxidant effect can shape WNT activity, since oxidative stress is known to control how stable β-catenin is inside cells [36]. Putting this together, it looks like VitC could strengthen β-catenin activity in both bone and immune cells. That might mean less bone loss and a better handle on inflammation. For DKK1 and Wnt5a, though, there is no direct evidence yet. DKK1 blocks WNT and is tied to joint damage in RA, and Wnt5a drives aggressive fibroblast-like synoviocytes in RA joints [37]. Whether VitC can influence those two specifically is still an open question. Overall, VitC seems to be a likely biological modulator of WNT-related mechanisms in RA, mainly through its β-catenin-activating and antioxidant properties, though direct proof in patient-derived joint cells is lacking. This is a new path for more studies.

Concluding, current findings suggest that VitC has potential value both in reducing the risk of RA and in influencing disease activity. While the available data support its antioxidant and immunomodulatory effects, larger and more targeted randomized trials are still needed to confirm its therapeutic role and establish practical recommendations for clinical use.

## 4. Sjögren’s Disease

SD is a chronic systemic autoimmune disorder characterized primarily by lymphocytic infiltration of exocrine glands, leading to dryness of the eyes and mouth, and it may also present with systemic manifestations affecting joints, lungs, and other organs. Recent reviews emphasize its heterogeneous presentation and highlight oxidative stress and immune dysregulation as central to its pathogenesis [38,39].

Xerostomia and keratoconjunctivitis sicca are symptoms of SD. It is becoming more widely acknowledged that oxidative stress plays a role in both ocular surface damage and glandular dysfunction. High levels of VitC are found in salivary secretions and the tear film, where it promotes vitamin E recycling and epithelial defence [40,41].

Some evidence for the protective role of dietary VitC comes from epidemiological data. Higher VitC intake was linked to lower odds of primary SD in a cohort following a Mediterranean diet, but this relationship diminished after full adjustment [42]. Likewise, dietary evaluations of women with primary SD revealed that, especially for supplement users, VitC intake was largely within recommended ranges [43].

Biochemical studies confirm a redox imbalance in primary SD. Increased lipid peroxidation and nitric oxide production, coupled with reduced antioxidant enzyme activity, have been documented and correlate with higher inflammatory cytokine levels and disease activity scores [44]. These findings support a rationale for antioxidant supplementation as an adjunct to standard management.

Clinical intervention studies remain limited. In patients with dry eye, a frequent and clinically significant manifestation of SD, oral antioxidant supplementation containing vitamins A, C, and E demonstrated improvements in tear stability and ocular surface staining compared with placebo [45]. Reviews and consensus statements on nutritional support for dry eye similarly list VitC as a potentially useful component of antioxidant regimens, though the evidence base is heterogeneous [41,46,47]. On the salivary side, VitC tablets are widely employed in clinical research to stimulate salivary flow for diagnostic purposes. A recent model for non-invasive diagnosis of primary SD incorporated stimulated saliva using VitC tablets, underscoring its accepted role in salivary testing protocols rather than as a disease-modifying therapy [48].

Systematic reviews and meta-analyses in dry eye cohorts further support the role of oxidative stress in disease pathophysiology, reinforcing the biological plausibility for VitC supplementation [49]. However, robust randomized controlled trials (RCTs) specifically evaluating VitC in SD are lacking. Current evidence therefore supports VitC primarily as a dietary factor and as part of combined antioxidant approaches, with limited data on its independent therapeutic effect.

## 5. Type 1 Diabetes

VitC has been shown to play an important role in the prevention and treatment of T1D. A meta-analysis revealed a positive correlation between dietary VitC intake and VitC serum levels in people with pre-diabetes and diabetes [50]. The authors also suggested that patients with diabetes with lower and deficient VitC serum levels had a shorter median survival compared with those with normal serum levels. Another study demonstrated that VitC may improve endothelial dysfunction in T1D, and may mediate an endothelial resistance to the action of glucagon-like peptide 1 (GLP-1) [51]. Furthermore, VitC infusion (30 mg/min for 2 h) reduced oxidative stress and inflammation in T1D, and it may exert a protective effect during acute hypoglycemia [51]. In patients with hypertension, intradermal microdialysis of VitC improved reflex cutaneous vasodilation through both nitric oxide–dependent and non-nitric oxide–dependent mechanisms [52]. This effect has been attributed to VitC’s ability to increase nitric oxide bioavailability by reducing oxidative stress and superoxide-related nitric oxide inactivation, and improving the activity of nitric oxide synthase through enhanced bioavailability of its cofactor tetrahydrobiopterin [3]. Additionally, the decrease in microvascular endothelium-dependent function in patients with lower VitC levels is thought to play a role in protecting against the development of cardiovascular disease in T1D [52]. VitC supplementation has also been shown to enhance cutaneous vasodilation in human skin [52].

Beyond its role in stem cell differentiation, osteoblast lining, collagen synthesis, bone formation, and various other biological processes, VitC is also transported into bone marrow stromal cells (BMSCs) and bone via sodium-dependent VitC transporter 2 (SVCT2) [53]. Although the mechanisms underlying the initiation of secondary osteoporosis following T1D are not fully understood, it is known that in BMSCs, SVCT2 is regulated by oxidative stress and steroid hormones, and that T1D induces oxidative stress and regulates SVCT2 in the bone and bone marrow environment [53]. This transporter facilitates in vitro differentiation of BMSCs into osteoblasts. However, more detailed studies focused on SVCT2 regulation in the bone and bone marrow environment of T1D are required [53].

The use of an antioxidant, such as VitC, in patients with T1D can help normalize endothelial dysfunction when combined with insulin. However, adding VitC to telmisartan therapy had no significant additional effect on the levels of nitrotyrosine plasma or endothelial function [54].

It is suggested that dietary factors during the fetal period, infancy, and childhood may trigger, inhibit, or modify the autoimmune processes that lead to T1D. One study found that dietary VitC supplementation was associated with a decreased risk of T1D, yet prospective studies assessing the effects of VitC in the disease process of T1D are scarce. VitC is an essential micronutrient that must be obtained from the diet, and it may have a supportive function against T1D due to its antioxidant properties. Nevertheless, maternal intake of VitC during pregnancy does not have an association with the risk of developing islet autoimmunity [55].

VitC supplementation did not have a significant effect on preeclampsia prevention in the overall T1D cohort; however, experimental data in diabetic animals suggested that VitC supplementation can ameliorate the risks associated with T1D [56]. A human controlled intervention study in pregnant women with T1D found a lower risk of premature birth in women receiving VitC and E supplementation, but VitC and VitE supplementation failed to prevent preeclampsia in women with T1D and a high-risk pro-angiogenic haptoglobin genotype. VitC supplementation may be beneficial in women with a low antioxidant status at baseline in terms of preeclampsia prevention [56].

The authors of another study suggested that VitC and VitE supplementation have a positive effect on lipoprotein profile and vibration perception threshold. They found that combined treatment with VitC and VitE for 4 weeks reduced the urinary albumin excretion rate (UAER) in patients with type 2 diabetes and persistent micro/macroalbuminuria. VitC supplementation was also found to restore impaired endothelium-dependent vasodilatation in patients with T1D. However, VitC administration had no effect on renal function in patients with nephropathy [57].

Taken together, these findings suggest that there are potential benefits of VitC supplementation for T1D.

## 6. Crohn’s Disease

CD is a chronic, immune-mediated inflammatory disorder of the gastrointestinal tract. The role of VitC in the course and progression of CD has yet to be determined. However, VitC deficiency is a common symptom of CD and has been linked to the development of serious health complications, including scurvy. Although uncommon today, it remains a recognized complication in CD, largely due to reduced fruit and vegetable intake, malabsorption, and restrictive diets. Several case reports have highlighted that VitC deficiency in CD can manifest with nonspecific or misleading clinical symptoms, including mucosal ulcerations, gingival bleeding, arthralgia, or even findings mimicking vasculitis [58,59]. In one report, scurvy was confirmed in a patient with CD manifesting as extensive oral lesions, with rapid improvement following VitC supplementation [60]. Another recent case described spontaneous hematomas in a CD patient adhering to a restrictive diet, where severe VitC deficiency was ultimately identified as the underlying cause [61]. These observations emphasize the importance of considering micronutrient deficiencies, including VitC, in CD patients with unusual or unexplained symptoms.

VitC has been hypothesized to play an important role in treating CD by reducing the oxidative stress [62,63]. Moreover, increased ROS and decreased status of antioxidant defences, including VitC, have been observed in Crohn’s strictures [64]. This oxidative stress has been linked to the development of CD, and it is further evidenced by the presence of 8-hydroxyguanine (8-OHDG) in the inflamed part of the bowel [64]. 8-OHDG is a product of DNA modifications caused by oxidative stress: It is formed as a result of DNA damage caused by ROS [64]. These findings point to the role of ROS in the pathogenesis of CD and suggest that oxidative stress can be a major factor in the development and progression of CD [64]. Low VitC contents have been observed in patients with CD [64]. In addition, vitamin deficiencies in patients with CD may be due to the disease itself or reduced dietary intake of VitC. Malnutrition and weight loss are common in patients with CD. They result from reduced dietary intake, malabsorption, diarrhea, and oxidative stress. Nutritional status is often already impaired at the time of diagnosis in patients with inflammatory bowel disease [65].

Another study was conducted to assess the VitC status of patients with CD, and the results revealed decreased serum concentrations of beta-carotene, VitC, VitE, as well as folic acid [66]. Additionally, researchers have reported that patients with CD patients have significantly lower VitC levels than healthy controls [65]. Furthermore, the VitC status of patients with CD remains unchanged until the disease has become severe [67]. Low VitC serum concentrations have also been found in patients with CD [68].

Although the potential side effects of taking VitC supplements in patients with CD are not well-defined, physicians have recommended the use of VitC supplementation in addition to anti-inflammatory medications, disodium cromoglycate, and sulfasalazine [69] to prevent or reverse VitC deficiency [65]. This is also important for patients with CD who are prescribed steroids and corticosteroids, the lifetime use of which may increase the risk of VitC deficiency [70,71]. As such, nutritional deficiency screening is recommended for patients with CD to improve dietary intake and prevent VitC-deficient conditions [72].

## 7. Hashimoto’s Thyroiditis

HT is the most prevalent autoimmune thyroid disorder, affecting millions of individuals throughout the world [73]. Characterized by the immune system’s attack on the thyroid gland, HT leads to chronic inflammation, thyroid dysfunction, and various clinical manifestations [74]. Despite the advancements in understanding the disease, effective treatments remain limited. Recent studies have indicated the potential therapeutic role of VitC in managing HT, shedding light on its anti-inflammatory, antioxidant, and immunomodulatory properties [75]. However, further research is needed to confirm these findings.

One of the studies on patients with autoimmune thyroiditis indicated that VitC showed no significant effect on the levels of thyroid stimulating hormone (TSH) and thyroglobulin antibody (Tg-Ab) in the VitC group compared with the control group. However, the thyroid peroxidase antibodies (TPO-Ab) levels decreased significantly after VitC administration, demonstrating the vitamin’s antioxidant benefits on thyroid-specific antibodies [76]. The positive effects of VitC on TPO-Ab may suggest that it could be a helpful adjunct treatment for HT. In another study, the authors demonstrated that the use of antioxidants—including VitC, among others, significantly elevated the blood levels of hormones produced by the thyroid, namely triiodothyronine (T3) and thyroxine (T4), in rats, which could be an effect of direct participation of antioxidants on the thyroid gland or on the activity of deiodinase enzyme [77]. In their meta-analysis of dietary nitrate and nitrite intake in man, Ward et al. [78] observed that the association between higher nitrate exposure and thyroid cancer risk was more pronounced among individuals with lower VitC intake, suggesting that VitC status may influence susceptibility. More recently, an umbrella review of micronutrients and thyroid cancer found no consistent evidence that supplementation with vitamins, including VitC, reduces thyroid cancer risk [79]. Together, these findings indicate that while VitC may modify risk associated with environmental exposures such as nitrates, current data do not support a direct protective effect of VitC supplementation against thyroid cancer.

Complementary evidence indicates links between VitC and thyroid function at a population and genetic level. Among U.S. adults, greater VitC intake was associated with lower total T4 and nonlinear relations with FT4 and the FT3/FT4 ratio [80]. Mendelian-randomization analyses suggest a protective causal effect of higher circulating VitC on autoimmune hypothyroidism [81]. Moreover, metabolic profiling in euthyroid HT has identified biomarkers sensitive to antioxidant vitamins (including VitC), consistent with redox imbalance even before overt hypothyroidism [82].

Recent data suggest that VitC intake and circulating levels may influence disease expression in HT. In a cross-sectional analysis of NHANES 2007–2012, individuals with HT in the highest quartile of total VitC intake had significantly lower odds of hypothyroidism than those in the lowest quartile (adjusted OR ≈ 0.40) [83].

## 8. Periodontitis

PD is a chronic inflammatory disease characterized by progressive destruction of the periodontal ligaments and alveolar bone. It results from dysregulation of the host immune response to bacterial biofilm, where oxidative stress is a key mechanism [84,85]. ROS stimulate the expression of pro-inflammatory cytokines, chemokines, and matrix metalloproteinases, hence amplifying tissue injury and making oxidative imbalance a predictor of severity [86,87].

VitC deficiency destabilizes the periodontal ligament, impairs healing and enhances sensitivity to tissue destruction. Clinical and experimental evidence uniformly show that insufficient levels of VitC are associated with greater inflammation and loss of tissue [88,89].

This association is supported by more recent work. Cross-sectional data reveal that PD patients have significantly lower levels of serum and salivary VitC compared with their healthy counterparts [90,91]. Observational data from Australian populations also confirm that plasma VitC deficiency is associated with advanced disease [91]. These findings demonstrate the reproducibility of this association in different populations and highlight the need to consider the nutritional status when assessing periodontal risk.

Intervention trials suggest that supplementation with VitC can provide modest clinical benefit. Sub-analysis of a randomized trial as a case-series showed that daily supplementation with VitC, with or without flavonoids, improved levels of antioxidants in the systemic circulation and reduced gingival inflammation, but the incremental value over non-surgical therapy was modest [92]. Dietary surveys using NHANES data also indicated that low VitC intake was strongly correlated with higher PD prevalence [93], consistent with earlier evidence that malnutrition accelerates disease progression [94].

Systematic reviews and meta-analyses also concur with these results. A 2024 review arrived at a conclusion that VitC supplementation improves periodontal disease parameters such as gingival index, bleeding on probing, and probing depth but heterogeneity of study design and baseline nutritional status limited the quality of inferences [95]. Companion reviews have emphasized that antioxidants, including VitC, contribute to the prevention of oxidative stress and inflammatory burden, which may enhance healing and treatment response [96,97].

The interaction between VitC and systemic disorders deserves attention. VitC can potentially act synergistically in combination with other antioxidants like vitamin E and polyphenols, whereas systemic disorders like diabetes, obesity, and smoking tend to increase oxidative stress and modify VitC needs [84,85]. Management of these factors by concurrent nutritional and periodontal therapies could potentially maximize clinical efficacy.

Overall, VitC deficiency is common in PD patients and inextricably associated with more advanced clinical expression. Some outcomes are aided by supplementation, particularly with addition to conventional therapy, but efficacy varies with dosage, initial status, and patient risk profile. Provision of adequate dietary VitC is a safe and reasonable practice to ensure periodontal health, since further trials are needed to define optimal regimens and identify likely subgroups to benefit.

## 9. Pernicious Anemia

PA is a rare autoimmune disorder that is related to abnormal absorption of vitamin B12 (VitB12) and leads to megaloblastic anemia. Although deficiency of VitB12 is the hallmark of PA, findings suggest that VitC plays an important role in hematologic as well as systemic manifestations. Autophagy, iron uptake, folate metabolism, and mitigation of oxidative stress are VitC-mediated. But increased doses are associated with thrombocytopenia and an increased rate of bruising [98]. PA accounts for 20–50% of adult VitB12 deficiency [99], and VitC metabolism disorders are increasingly being recognized as being of clinical relevance in this respect [100].

Current case reports and small series indicate that severe VitC deficiency is able to further aggravate the hematological profile in PA. Megaloblastic or hemolytic anemia was observed in patients, which was cured after VitC supplementation [101,102]. These results suggest that deficiency of VitC can further increase macrocytosis, impair red blood cell maturation, and increase hematologic manifestations in patients with PA.

Gastric pathology plays a central role in this interaction. Autoimmune gastritis, the pathogenesis of PA, is characterized by chronic hypochlorhydria, which not only damages cobalamin absorption but also destabilizes intragastric VitC. Current studies demonstrate that plasma and gastric juice levels of VitC are profoundly suppressed in autoimmune gastritis patients. Supplementation normalizes these levels but is blunted in hypochlorhydric individuals [103]. These findings underscore the gastric physiology-antioxidant metabolism cross-talk and underscore the need to monitor VitC status in PA patients.

More extended nutritional evaluations still prove that patients with chronic atrophic autoimmune gastritis often suffer from deficits of various micronutrients, including VitC, iron, and folate, and also cobalamin [104]. Such deficiencies can cause not only hematologic dysfunction, but also extraintestinal symptoms, e.g., fatigue, healing impairment of wounds, and increased oxidative stress burden.

In general, the information suggests that VitC deficiency can worsen the clinical course of PA and quality of life. Parenteral VitB12 supplementation remains the cornerstone of treatment, but evaluation of VitC status can identify individuals who may benefit from adjunctive supplementation. Physicians must, however, avoid side effects, and dosing regimens must be individualized according to patient age, comorbidities, and overall health status [98].

## 10. Antiphospholipid Syndrome

APS is an autoimmune clotting disorder. Antiphospholipid antibodies (aPL) switch on the vessel lining, platelets, and monocytes; complement and neutrophil extracellular traps (NETs) then amplify coagulation and vascular injury. Across lab and patient studies, one theme keeps popping up: oxidative stress. People with aPL/APS show more lipid peroxidation (e.g., F2-isoprostanes), more ROS, and a redox-driven rise in monocyte tissue factor (TF) changes that can tip the balance toward thrombosis [105,106,107,108]. Vitamin C is a water-soluble antioxidant that helps the vessel lining keep nitric oxide (NO) available and can dial down overactive white blood cells (including NET-forming neutrophils) in non-APS settings; in humans, a randomized trial also showed it can restore flow-mediated vasodilation during acute inflammation [109,110,111]. Together, these points make a reasonable case for testing vitamin C in APS, even though disease-specific outcome data are still missing [109,110,111].

Direct interventional evidence in APS is limited to small, biomarker-focused work. The most specific human signal is a supplementation study in aPL-positive patients receiving high-dose vitamin E plus vitamin C for several weeks, which reduced urinary F2-isoprostanes and monocyte TF antigen/activity markers linked to oxidative injury and a procoagulant state without assessment of clinical endpoints [112]. Contemporary reviews of APS redox biology cite this as proof-of-principle that antioxidant strategies can shift pathogenic biomarkers but emphasize the absence of outcome data and do not recommend vitamin C as therapy for APS at present [105,106,107,113,114]. In parallel, growing literature connects APS with endothelial dysfunction and extracellular vesicles (EVs) of endothelial and platelet origin, which are increased in APS and obstetric APS and associate with dis-ease features; although vitamin C can influence endothelial redox tone and EV biology in other settings, an APS-specific test of vitamin C on EVs is still lacking [115,116,117]. NETs are now recognized contributors to APS thrombosis; antioxidants, including vitamin C, can sup-press NET formation in experimental systems and some human models, but APS-targeted trials are not yet available [110,111].

In sum, vitamin C has a sound mechanistic rationale in APS based on redox biology, endothelial function, and innate immune modulation, and a small aPL-positive study suggests favourable biomarker effects when combined with vitamin E [106,107,108,109,110,111,112]. However, no randomized trials have shown that vitamin C reduces clinical events (thrombosis or obstetric morbidity), and current reviews do not endorse it as therapy. If considered at all, vitamin C should be adjunctive, never replacing anticoagulants, and investigated in phase II studies that link redox/EV/NET biomarkers to clinical outcomes, with careful phenotyping (thrombotic vs. obstetric APS), aPL risk profile (including triple-positive subsets), and standardized background anticoagulation [105,106,107,112,113,114,115,116,117].

## 11. Alzheimer’s Disease

AD is a progressive neurodegenerative disorder characterized by memory loss, cognitive decline, and functional impairment. Its pathogenesis is complex and multifactorial, involving amyloid-β accumulation, tau hyperphosphorylation, oxidative stress, and mitochondrial dysfunction. Increasingly, researchers have also emphasized autoimmune features of AD. Weaver [118,119] proposed that β-amyloid (Aβ) may act as an immunopeptide, triggering maladaptive immune responses against host neurons. Arshavsky [120] suggested that disruption of the blood–brain barrier permits autoreactive immune activity, while Severini et al. [121] identified the NLRP3 inflammasome as a mediator of innate immune activation. These findings suggest that, although AD is fundamentally a neurodegenerative disorder, autoimmune mechanisms contribute to its progression and amplify neuroinflammatory damage.

Within this context, antioxidant defences have received particular attention. Vitamin C (VitC) is a major antioxidant in the central nervous system, where it maintains redox homeostasis, supports neurotransmitter synthesis, and protects neurons from oxidative injury. A quantitative meta-analysis confirmed that plasma VitC levels are significantly lower in AD patients compared with controls [122]. Population-level data extend this observation: in a national U.S. cohort, higher baseline serum VitC concentrations were associated with lower AD-related mortality during long-term follow-up, although excessively high levels did not confer additional benefit [123].

VitC intake also appears to influence cognitive function. An NHANES-based analysis identified a non-linear dose–response relationship, with higher VitC intake linked to improved performance in processing speed and memory, though the benefit plateaued at higher intakes [124]. Similarly, a 2025 meta-analysis of prospective cohorts reported that dietary VitC intake ≥75 mg/day was associated with a reduced risk of AD, whereas supplemental VitC did not consistently provide protection [125]. These findings suggest that adequate VitC derived from diet may contribute to cognitive resilience and risk reduction.

Mechanistic evidence supports these associations. VitC has been shown to modulate oxidative stress, homocysteine metabolism, and inflammatory signalling, as well as to interact with amyloid-β aggregation and tau phosphorylation, central processes in AD pathogenesis [126]. Importantly, clinical and experimental studies have documented elevated markers of oxidative stress in AD brains, accompanied by reduced antioxidant defences [127]. Furthermore, increased lipid peroxidation and protein oxidation have been observed in both plasma and cerebrospinal fluid of AD patients, correlating with disease progression [128]. These findings reinforce the hypothesis that an imbalance between oxidative burden and antioxidant capacity accelerates synaptic dysfunction and neuronal loss.

Other studies have pointed to genetic and epigenetic regulation, lifestyle factors, and comorbidities as additional modifiers of oxidative stress and AD progression [129,130]. Together, these data indicate that VitC insufficiency may contribute to the oxidative and inflammatory environment characteristic of AD, while adequate intake and circulating levels are associated with improved cognitive function and reduced risk or progression of the disease.

## 12. Conclusions

Vitamin C (VitC) has emerged as a potentially important modulator of immune and inflammatory pathway in immune-mediated (including autoimmune) diseases. Low VitC status has been linked to increased levels of oxidative stress, increased disease activity, and impaired tissue repair in numerous conditions. Supplementation trials, though heterogeneous in design, suggest possible gains in reversing redox imbalance, regulating immune function, and preserving tissue integrity. These findings represent a solid biological rationale for the consideration of VitC as an adjunct ingredient in the treatment of immune-mediated disease.

Table 1 presents the evidence level between different immune-mediated disorders. While consistency and study quality differ, moderate evidence is present in such diseases as RA, PD, and AD, with earlier but less strong data for others. This pattern shows the practicality of VitC under certain conditions and areas most under intense research required.

VitC may be regarded as a safe, easily available adjunct that could possibly supplement existing therapies but not substitute them. It may be of particular benefit in the context of deficiency, severe oxidative stress, or the presence of coexistent risk factors. RCTs with ideal standardized design and clinical endpoints are anticipated in the future to determine ideal dosing regimens and also to determine patients likely to benefit from VitC. With integration with current knowledge and ongoing research, VitC can be a potential adjunct approach to sustaining improved results and quality of life for autoimmune and other immune-mediated disease patients.

## Figures and Tables

**Table 1:** Strength of evidence for vitamin C (VitC) in autoimmune/immune-mediated diseases.

Disease	Evidence Type	Findings	Strength of Evidence *
Multiple sclerosis	Observational, small clinical studies	Reduced serum VitC; some improvement in oxidative stress; limited trial data	Limited
Rheumatoid arthritis	Population cohorts, experimental, small RCTs	Inverse associations; antioxidant and immunomodulatory effects; ongoing trials	Moderate
Sjögren’s disease	Dietary studies, small interventions	Antioxidant rationale; modest benefits in dry eye; no disease-modifying data	Limited
Type 1 diabetes	Animal models, small human interventions	Endothelial and oxidative improvements; no consistent glycemic benefit	Limited
Crohn’s disease	Case reports, biochemical analyses	Frequent deficiency; links to oxidative stress; supplementation prevents scurvy	Limited
Hashimoto’s thyroiditis	Observational, meta-analyses, animal studies	Possible antibody reduction; mixed population data; no strong RCTs	Limited –Moderate
Periodontitis	Cross-sectional, clinical interventions	Consistent link with deficiency; supplementation modestly supports standard therapy	Moderate
Pernicious anemia	Case reports, mechanistic studies	Deficiency worsens hematologic profile; supplementation helpful but not curative	Limited
Antiphospholipid syndrome	Small biomarker trials, mechanistic rationale	Antioxidant effects demonstrated; no outcome-based clinical data	Limited
Alzheimer’s disease	Observational cohorts, meta-analyses	Lower VitC in patients; dietary intake linked to reduced risk; supplementation less consistent	Moderate

* Strength of evidence graded qualitatively as Limited, Moderate, or High, reflecting the consistency, quality, and size of available studies. No condition currently has high-level evidence.

## Data Availability

No new data were created or analyzed in this study. Data sharing is not applicable to this article.

## Abbreviations

The following abbreviations are used in this manuscript:

AD	Alzheimer’s disease
APS	antiphospholipid syndrome
CD	Crohn’s disease
ECM	extracellular matrix
HT	Hashimoto’s thyroiditis
MS	multiple sclerosis
PA	pernicious anemia
PD	periodontitis
RA	rheumatoid arthritis
RCT	randomized controlled trial
ROS	reactive oxygen species
SD	Sjögren’s disease
T1D	type 1 diabetes
VitC	vitamin C
